# 不同因素对pⅢa/N2期非小细胞肺癌患者预后的影响

**DOI:** 10.3779/j.issn.1009-3419.2010.08.06

**Published:** 2010-08-20

**Authors:** 真榕 张, 德若 刘, 永庆 郭, 彬 石, 之乙 宋, 燕雏 田

**Affiliations:** 100029 北京，北京大学中日友好医院胸外科 Department of Thoracic Surgery, China-Japan Friendship Hospital, Peking University, Beijing 100029, China

**Keywords:** 肺肿瘤, 转移, 预后, Lung neoplasms, Metastasis, Prognosis

## Abstract

**背景与目的:**

目前对pⅢa/N2期非小细胞肺癌患者所采取的治疗方法尚不一致。本研究旨在评价不同影响因素与pⅢa/N2非小细胞肺癌患者预后的相关性。

**方法:**

回顾性分析1998年1月-2004年5月133例非小细胞肺癌患者经以外科干预为主的综合治疗后的无瘤生存期和5年生存率。研究因素包括年龄、性别、跳跃转移、淋巴结转移站数、手术类型、病理分型、辅助治疗等。应用SPSS 16.0软件统计生存率。

**结果:**

133例pⅢa/N2期入组患者总的5年生存率为32.33%，单站淋巴结转移亚组与多站淋巴结转移亚组的5年生存率分别为39.62%和27.50%；临床N0-1分期（cN0-1）亚组与临床N2分期（cN2）亚组的5年生存率分别为37.78%和20.93%。*Cox*回归分析显示：淋巴结转移站数（*P*=0.013, OR=0.490, 95%CI: 0.427-0.781）及cN0-1（*P*=0.009, OR=0.607, 95%CI: 0.372-0.992）与N2患者预后呈正相关。

**结论:**

非小细胞肺癌的cN分期、淋巴结转移站数目与pⅢa/N2期患者的预后呈正相关；在严格入组条件下，可以对选择性pⅢa/N2患者进行以外科治疗为主、联合辅助治疗的综合治疗，并可获得较满意的长期生存率。

## Abstract (en)

**Background and objective:**

Thoracic surgery hasn't got consensus on therapy of pⅢa/N2 non-small cell lung cancer (NSCLC). The aim of this study is to identify prognostic factors in NSCLC with N2 nodal involvement.

**Methods:**

A retrospective review of disease free survival and 5-year survival for NSCLC patients who underwent primary surgical resection without neoadjuvant chemotherapy was performed. Between January 1998 and May 2004, 133 patients were enrolled. Several factors such as age, sex, skip metastasis, number of N2 lymph node stations, type of resection, histology and adjuvant therapy were recorded and analyzed. SPSS 16.0 was used for calculating survival.

**Results:**

Overall 5-year survival for 133 patients was 32.33%, and 5-year survival for single N2 station and multiple N2 station sub-group were 39.62% and 27.50%, respectively. 5-year survival for cN0-1 and cN2 sub-group were 37.78% and 20.93%, respectively. *Cox* regression analysis revealed that number of N2 station (*P*=0.013, OR=0.490, 95%CI: 0.427-0.781) and cN status (*P*=0.009, OR=0.607, 95%CI: 0.372-0.992) were two favorable prognostic factors of survival.

**Conclusion:**

Number of N2 station and cN status are two favorable prognostic factors of survival. In restrict enrolled circumstances, after combined therapy made up of surgery and postoperative adjuvant therapy have been performed, satisfied survival can be achieved.

肺癌是世界上发病率最高的恶性肿瘤之一，80%为非小细胞肺癌（non-small cell lung cancer, NSCLC）。国外研究^[1, 2]^显示，NSCLC中约30%的N2期患者5年生存率为10%-15%。淋巴结有无转移是判断肿瘤复发和预后的重要因素之一。Detterbeck^[3]^认为可以把pⅢa/N2期患者分成具有不同预后的亚组。目前很多医院对pⅢa/N2期病例采用包括新辅助治疗在内的综合治疗模式，然而2009年美国国家抗癌联盟^[4]^（National Care Comprehensive Network, NCCN）对于pⅢa/N2期肺癌的治疗并未给出结论，并且对于不同亚组的治疗方式也无进一步阐述。本研究通过对133例N2期NSCLC患者的总结，对不同亚组的N2患者的预后进行分析。

## 1 材料与方法

### 1.1 临床资料

1998年1月-2004年5月就诊于中日友好医院胸外科的NSCLC患者共685例，其中pⅢa/N2期211例，本组实验对象为符合入组标准的133例pⅢa/N2患者，其中，男性64例，女性69例; 平均年龄57.61岁（37岁-78岁）; 其中101例行肺叶/复合肺叶切除，6例行袖状肺叶切除，26例行全肺切除（表 1）。

**1:** 入组133例患者的临床资料 Clinical characteristics of 133 enrolled patients

Characteristics	*n* (%)
Age	(years)
≥65	32 (24.06%)
< 65	101 (75.94%)
Gender	
Male	64 (48.12%)
Female	69 (51.88%）
Operation	
Lobar/combined lobar resection	101 (79.70%)
Pneumonectomy	26 (16.54%)
Sleeve resection	6 (4.51%)
T stage	
T1	13 (9.77%)
T2	79 (59.40%)
T3	41 (30.83%)
Operation side	
Right	78 (58.65%)
Left	55 (41.35%)
Metastasis (station)	
Single station	53 (39.85%)
Multiple stations	80 (60.15%)
Histology	
Squamous	56 (42.11%)
Adenocarcinoma	67 (50.38%)
Others	10 (57.52%)
cN stage	
cN0-1	90 (67.67%)
cN2	43 (32.33%)
Skip metastasis
Yes	41 (30.83%)
No	92 (69.17%)
Adjuvant therapy	
Radiochemotherapy	75 (56.39%)
Chemotherapy (CT)	58 (43.61%)

### 1.2 方法

入组标准：病理诊断为pⅢa/N2期NSCLC; 术前诊断cN0-2，术前未行新辅助治疗; 术中完成R0切除及系统性淋巴结清扫术; 术后3周-6周内行辅助治疗。排除标准：不符合上述入组标准的肺癌患者; 失访或非疾病相关性死亡（图 1）。

**1:**
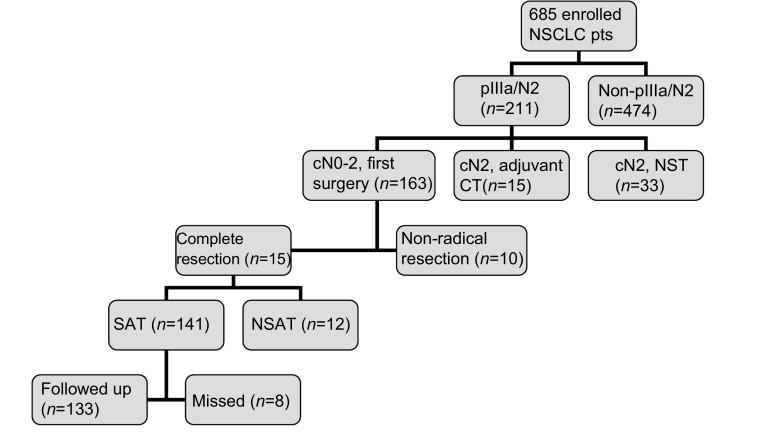
入组筛选过程 Enrolled process. NSCLC: non-small cell lung cancer; CT: chemotherapy; NST: non surgical treatment; SAT: standard adjuvant therapy; NSAT: non standard adjuvant therapy.

术前常规行胸部CT、上腹部CT、腹部B超、锁骨上B超、气管镜、头颅MRI、骨扫描进行分期，对于术前依靠以上方法无法准确判断临床分期的患者应用PETCT; 术前未进行纵隔镜分期; 手术采用常规剖胸手术，手术方式：肺叶切除、复合肺叶切除、袖状肺叶切除、全肺切除，同时行系统性淋巴结清扫术。术中完成R0切除，淋巴结清扫范围：如行右肺手术，常规清扫1R、2R、3、4R、7、8R、9R、10R-13R组淋巴结，如行左肺手术，常规清扫1L、2L、3、4L、5L、6L、7、8L、9L、10L-13L组淋巴结，1^st^与2^nd^组淋巴结共同送检，12^th^与13^th^组淋巴结随所切除肺叶共同送检; 术中病理回报淋巴结阳性，如能实现R0切除，行根治手术; 如无法完整切除，放弃手术或完成手术，但被剔除出组。

术后3周-6周内行以铂类为基础的化疗或序贯式放化疗; 133例入组患者中58例行化疗4周期-6周期，其中紫杉醇+顺铂/卡铂方案33例; 健择+顺铂/卡铂方案16例; 长春瑞滨+顺铂/卡铂方案9例。75例行序贯式化放疗，其中紫杉醇+顺铂/卡铂方案联合放疗49例; 健择+顺铂/卡铂方案联合放疗14例; 长春瑞滨+顺铂/卡铂方案联合放疗12例，所有化疗方案均为21天一个周期; 放疗剂量为50 Gy-60 Gy。

### 1.3 随访

术后前2年每3个月复查一次胸部CT，每半年复查一次头颅MRI、骨扫描; 第2年-第5年每半年复查一次胸部CT、头颅MRI、骨扫描。部分患者随访过程中根据病情变化行纤维支气管镜及其它检查。观察起始点为患者诊断为肺癌之日，随访终点为疾病相关性死亡或2009年5月1日，随访最短时间为5年。随访主要终点：疾病相关性5年生存率; 随访次要终点：无瘤生存率。R0切除：肉眼与镜下切缘均为阴性; cN0-1：胸部CT纵隔内淋巴结最大径不超过1 cm; 或PET/CT示SUV≤2.5;cN2：胸部CT纵隔内淋巴结最短径超过1 cm; 或者PET/SUV示SUV > 2.5。肺癌分期采用AJCC 1997年版^[5]^进行分期。

### 1.4 统计方法

应用SPSS 16.0软件进行统计分析; 连续变量用Mean±SD显示; 分离变量用比率显示; 分离变量组间比较应用独立样本*t*检验进行统计; 生存率应用*Kaplan-Meier*法进行统计; 多因素分析应用*Cox*回归模型进行统计; 以*P* < 0.05为差异具有统计学意义。观察指标包括：年龄、性别、术式、T分期、cN分期、病理类型、跳跃性转移、N2淋巴结转移站数、辅助治疗与5年生存率、无瘤生存期（progression-free survival, PFS）的关系。

## 2 结果

共133例pⅢa /N2患者入组，总体5年生存率为32.33%。单站N2淋巴结转移与多站N2淋巴结转移亚组的5年生存率分别为39.62%和27.50%（图 2）; cN0-1与cN2亚组的5年生存率分别为37.78%和20.93%（图 3）; 跳跃性与非跳跃性转移亚组的5年生存率分别为36.59%和30.43%（图 4）。单变异分析显示淋巴结转移站数、cN分期以及跳跃性转移与N2期NSCLC预后呈正相关，多变异分析显示淋巴结转移站数（*P*=0.013, OR=0.490, 95%CI: 0.427-0.781）、cN分期（*P*=0.009, OR=0.607, 95%CI: 0.372-0.992）与N2期NSCLC的预后呈正相关（表 2）。

**2:**
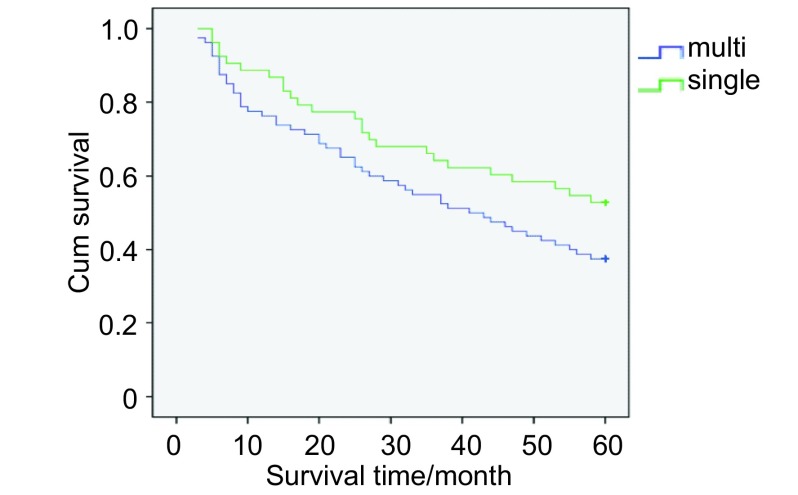
单站与多站转移亚组的生存曲线 Survival curves of single station metastasis group and multistation metastasis group. Single: single station metastasis group; multi: multi-station metastasis group.

**3:**
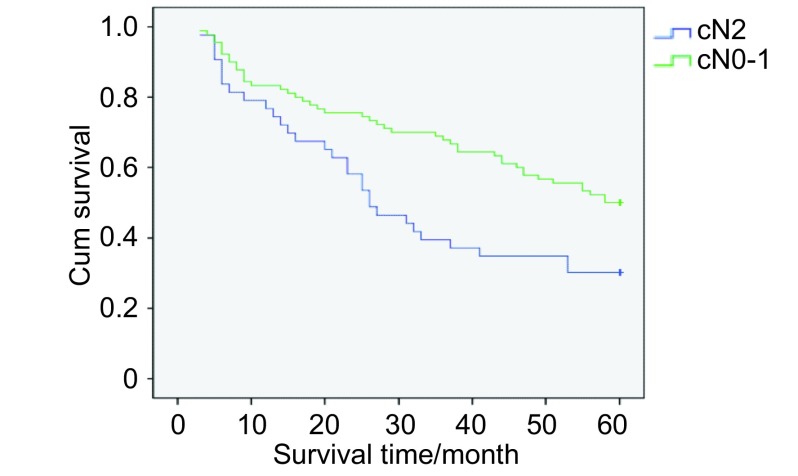
cN0-1与cN2亚组的生存曲线 Survival curves of cN0-1 group and cN2 group

**4:**
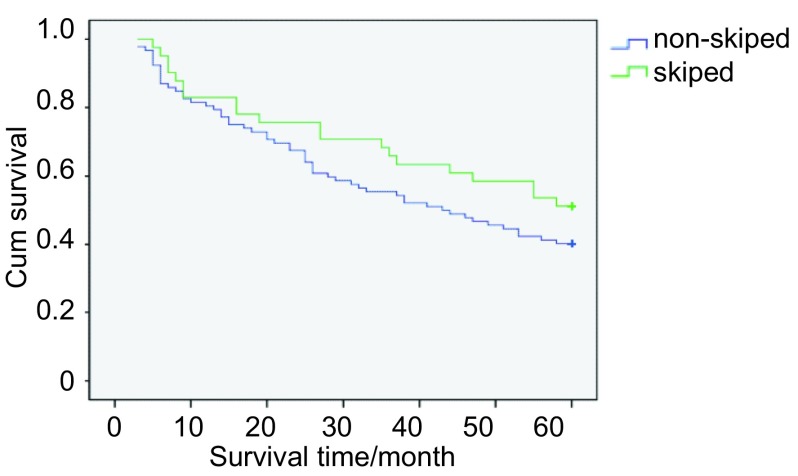
跳跃性与非跳跃性转移亚组的生存曲线 Survival curves of skip metastasis group and non-skip metastasis group. Skip: skip metastasis group; Non-skip: non-skip metastasis group.

**2:** 生存率相关的预后因子 Prognostic factors related to survival

Variants	Uni-variate *P* value	Multi-variate *P* value
Age	0.374	
Gender	0.512	
Operation	0.089	0.331
T stage	0.073	0.194
Operation side	0.221	
Metastasis station	0.011	0.013
Histology	0.239	
cN stage	0.005	0.009
Skip metastasis	0.048	0.140
Adjuvant chemotherapy	0.104	0.297

总体5年无瘤生存率为23.31%，单变异分析显示cN分期（*P*=0.025）、淋巴结转移站数、pT分期、辅助治疗与无瘤生存期呈正相关; 多变异分析显示淋巴结转移站数（*P*=0.003, OR=0.413, 95%CI: 0.342-0.723）、cN分期（*P*=0.040, OR=0.314, 95%CI: 0.112-0.671）、辅助治疗（*P*=0.014, OR=0.826, 95%CI: 0.573-1.291）与N2期NSCLC的预后呈正相关（表 3）。淋巴结转移分布见图 5。

**3:** 无瘤生存期相关的预后因子 Prognositc factors related to progression-free survival

Variants	Uni-variate *P* value	Multi-variate *P* value
Age	0.571	
Gender	0.233	
Operation	0.059	0.161
T stage	0.034	0.421
Operation side	0.409	
Metastasis station	0.001	0.003
Histology	0.709	
cN stage	0.025	0.040
Skip metastasis	0.348	
Adjuvant chemotherapy	0.008	0.014

**5:**
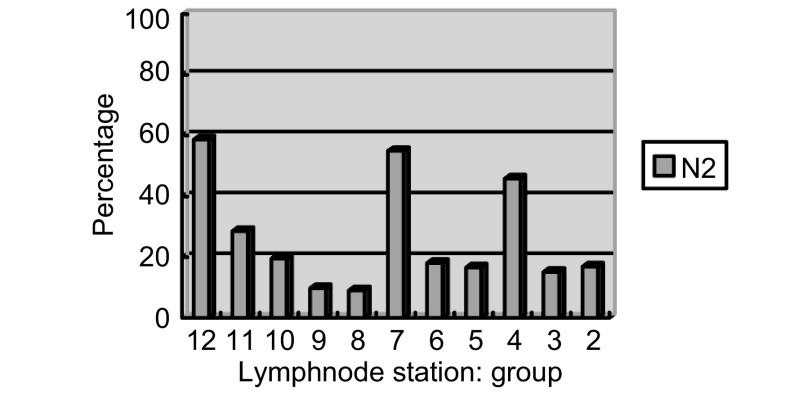
淋巴结转移分布图 Distribution of N2 lymph nodes

## 3 讨论

外科治疗是治疗NSCLC最有效的手段^[6]^。2009年NCCN^[4]^指出：以外科治疗为主的综合治疗模式已成为Ⅰ期、Ⅱ期NSCLC患者的标准治疗模式，然而对pⅢa/N2期患者的治疗方法仍然存在争议。

从结果中我们可以看到，淋巴结转移站数、cN分期与5年生存率、无瘤生存期具有相关性; 而辅助治疗仅与无瘤生存期具有相关性; 其它如年龄、性别、术式、术侧、组织学等因素与二者无相关性。

国外常规采用纵隔镜、PET/CT对cN2期肿瘤进行诊断，但Cerfolio进行的一项前瞻性研究^[7]^发现纵隔镜、超声内镜针吸活检（EUS guided fine needle aspiration, EUS-FNA）对于PET及CT诊断为N0患者的阳性率仅为2.9%和3.7%。Meyers等^[8]^研究发现对于CT和PET诊断为Ⅰ期NSCLC患者来说，隐匿性N2发生率为5.6%，因此对于cN0-1患者没有必要进行纵隔镜检查。本组术前未常规应用纵隔镜及PET/CT对肺癌进行分期，中日友好医院胸外科对于术前CT显示纵隔内淋巴结融合成团、与周围组织器官关系密切、考虑无法手术切除的病例（48例）首选新辅助化疗1个-2个周期，然后进行再评价，对于由cN2降为cN0-1的病例（15例）采用手术治疗; 对于未降期者（33例）继续行化疗或放化疗，但结果未包含在本实验中。

多个因素可影响N2期患者的预后，Ponn^[9]^的研究显示单站淋巴结转移、跳跃性转移、镜下或者薄膜内转移等与N2期患者的预后呈正相关; Detterbeck^[3]^的研究显示R0切除、跳跃性转移、单站N2转移、新辅助治疗的有效性、cN0-1等因素与N2期NSCLC的预后呈正相关，而性别、组织学类型、肿瘤位置、既往有无其它肿瘤史与预后无明显关系。

淋巴结转移复杂程度与肺癌恶性程度呈正比，与预后密切相关^[10]^。Cerfolio^[11]^的研究显示：单站N2及多站N2期NSCLC的5年生存率分别为40%和25%;Cox回归分析显示多站淋巴结转移与N2期NSCLC预后呈负相关; 在本试验中单站与多站N2亚组的5年生存率分别为39.62%和27.50%（图 2）; 多变异分析示单站N2淋巴结转移与预后呈正相关。单站与多站N2亚组的无瘤生存期分别为30.12%和18.75%（*P*=0.003），多变异分析示淋巴结转移站数与无瘤生存期呈正相关。多站N2亚组仅有15例患者术后5年未复发，说明多站N2转移恶性程度较高，术后复发倾向较高。

Saito^[12]^的研究显示：cN分期与pⅢa/N2期非小细胞肺癌预后有相关性，该研究发现了214例pN2病例，cN0、cN1、cN2、cN3亚组的5年生存率分别为30%、34%、14%和0%（*P*=0.04）。本实验结果显示cN0-1与cN2亚组的5年生存率分别为37.78%和20.93%（图 3）。多变异分析显示cN0-1与pⅢa/N2期患者的预后呈正相关。我们认为这与cN0-1亚组中单站N2淋巴结转移比率较高、全肺切除比率较低有关。实验结果同时显示cN0-1与cN2亚组的无瘤生存期分别为25.56%和18.60%，cN0-1与无瘤生存期呈正相关。

理解N2期的生物学特性对制定治疗方案有重要意义^[13]^。通常认为肺癌淋巴结转移遵循线性模式，Riquet^[14]^总结了731例pN2期NSCLC病例，结果显示29.2%N2期患者发生跳跃性转移; 跳跃性转移组的5年生存率为34.4%，多变异分析提示跳跃性转移与5年生存率呈正相关（*P* < 0.05）。Cerfolio^[11]^对142例患者进行总结后并未发现跳跃性转移与N2期患者的预后成正相关。在本实验中，跳跃性与非跳跃性转移亚组的5年生存率分别为36.59%和30.43%，但多变异分析显示跳跃性转移并非pN2患者的独立预后因子。

全肺切除术后并发症发生率较高，对患者术后生活质量影响较大，有研究^[14]^显示全肺切除是N2期肺癌预后的独立危险因素。袖状切除在一定情况下可以替代全肺切除，不但可实现肿瘤的根治术，同时可实现保护肺功能、改善术后生活质量的目的^[15]^。本实验组行全肺切除26例（右全肺4例，左全肺22例），袖状切除6例，肺叶/复合肺叶切除101例，围手术期并发症发生率为13.53%，其中心律失常8例，肺部感染4例，肺漏气5例，肺动脉栓塞1例。肺叶/复合肺叶切除、全肺切除、袖状切除亚组的5年生存率分别为33.66%、26.32%和33.33%，多因素分析并未显示术式是预后的独立危险因素。全肺切除组术后并发症发生率与其它两组相比稍低（11.54% *vs* 14.02%），但差异无统计学意义（*P*=0.61）; 我们认为全肺切除组5年内生存率较其余两组低的原因可能为：肿瘤T分期相对较晚（T3期19例）; 术后化疗相对较晚：22例全肺切除患者术后第5-6周进行化疗。因此我们认为对于IIIa/N2期病例而言，全肺切除是安全可靠的，如条件允许，亦可以选择袖状切除术。

有研究显示肿瘤的pT分期与预后呈密切相关，Riquet^[14]^的研究显示随着肺癌体积增大，N2患者的5年生存率逐渐下降。本实验虽然患者的生存率随着肺癌体积增大而下降（38.46%、34.18%和29.27%），但多因素分析并未提示肿瘤T分期与患者生存率相关（*P*=0.194），这与其它几项研究^[14, 16]^结果相同。

辅助化疗可以为大部分NSCLC带来生存获益，2007年ACCP指出：N2期肺癌术后应常规应用以铂类为基础的化疗^[6]^。一项回顾性研究^[17]^结果显示新的放疗技术可以降低并发症发生率，并给患者带来生存获益。本组对不同术后辅助治疗亚组进行比较，术后行化疗58例，放化疗75例，化疗与放化疗亚组相比，虽然5年生存率较低（29.31% *vs* 34.67%），但差异无统计学意义（*P*=0.104）。术后放化疗组11例发生放射性肺炎，其中7例需进行吸氧及药物治疗。化疗和放化疗亚组的无瘤生存期分别为18.97%和26.67%，多变异分析显示术后放化疗与无瘤生存期呈正相关（*P*=0.014）。因此我们认为虽然术后放疗对无瘤生存期有益，但放疗并发症发生率较高，且无法提高5年生存率，因此建议术后早期不必常规放疗，对于多站N2转移、肿瘤分化程度低、非R0切除的高危病例可选择放疗。

本研究中入组N2期NSCLC患者5年生存率较高的原因为：术前准确判断cN2病例的可切除性，无法完整切除者被剔除; 大部分病例为cN0-1期; 术前新辅助治疗者被剔除出组; 肺叶/复合肺叶切除比率高，全肺切除比例较低，部分病例行袖状切除术代替全肺切除术; 所有入组病例均为R0切除，并完成系统性淋巴结清扫术; 术后早期放化疗。本实验不足之处：本研究为回顾性分析; 切除淋巴结数目、阳性淋巴结数目未统计; 术前未常规应用PET和纵隔镜进行分期; 入组病例数较少。

总之，在严格入组标准条件下，仍然可以对选择性N2患者进行以外科治疗为主、联合辅助治疗的综合治疗，并获得较满意的长期生存率。
